# Cancer-related fatigue: benefits of information booklets to improve patients’ knowledge and empowerment

**DOI:** 10.1007/s00520-022-06833-w

**Published:** 2022-02-11

**Authors:** Martina E. Schmidt, Marlena Milzer, Cécile Weiß, Paul Reinke, Miriam Grapp, Karen Steindorf

**Affiliations:** 1 Division of Physical Activity, Prevention and Cancer, National Center for Tumor Diseases (NCT) and German Cancer Research Center (DKFZ), 69120 Heidelberg, Germany; 2 Psycho-Oncology Service, National Center for Tumor Diseases (NCT) and Department of General Internal and Psychosomatic Medicine, University Hospital Heidelberg, Heidelberg, Germany

**Keywords:** Fatigue management, Cancer survivorship, Quality of life, Health literacy, Patient education, Pamphlets

## Abstract

**Purpose:**

To investigate cancer patients’ knowledge and attitudes regarding fatigue and the potential benefits and acceptability of a brief information booklet.

**Methods:**

The CARPE DIEM study assessed knowledge and attitudes regarding fatigue in a diverse group of 50 cancer patients before (T0) and about one (T1) and four months (T2) after reading the booklet. At T1, participants additionally rated its usefulness.

**Results:**

At baseline, 37.5% of respondents did not know the term “fatigue” or what it meant. Those who already knew something about fatigue mainly had obtained their information from booklets, books, or articles (63.3%) and/or the internet (46.7%). Overall, knowledge gaps existed, particularly about potential fatigue treatment options and whether fatigue is an indicator of cancer progression. Furthermore, 56.4% felt poorly informed, and 46.1% reported feeling helpless in the face of fatigue. Lower knowledge at baseline was significantly associated with lower education and older age. At T1 and T2, there were significant improvements in several knowledge questions and attitudes. Patient-reported benefits included getting new information about fatigue (91.1%), awareness of not being alone with their problems (89.7%), taking appropriate actions (72.9%), and encouragement to talk about their fatigue with family/friends (55.3%) or with a health professional (52.7%).

**Conclusions:**

Specific gaps were identified in the provision of information and education for cancer patients about fatigue. A low-cost intervention asking to read a brief information booklet was associated with improved knowledge. This could be considered as a first step offered as part of a bundle of further efforts to improve knowledge and care of fatigue.

**Supplementary Information:**

The online version contains supplementary material available at 10.1007/s00520-022-06833-w.

## Introduction

Cancer-related fatigue is a common and very burdensome problem, defined as a distressing, persistent, subjective sense of physical, emotional, and/or cognitive tiredness or exhaustion related to cancer or cancer treatment that is not proportional to recent activity and interferes with usual functioning [1]. Although there is evidence for effective treatment options such as aerobic and resistance exercise, yoga, or psychosocial interventions [2–8], fatigue often remains unnoticed and untreated [9]. Clinical practice guidelines for the management of fatigue were published by the US National Comprehensive Cancer Network (NCCN) [2], the Canadian Association of Psychosocial Oncology (CAPO) [10], and the European Society for Medical Oncology (ESMO) [3]. As one part of comprehensive fatigue management, they all recommend informing and educating patients about fatigue, i.e., about its multifactorial nature, potential causes and influencing factors, and known patterns during and following cancer treatment. However, in a recent German survey among 2508 cancer survivors, more than half of the participants reported feeling poorly informed about fatigue. In addition, more than one-fifth of the participants believed that there is no way to alleviate fatigue. A remarkable number of patients further agreed with the statements that fatigue is a side effect that has to be accepted (40%) and that fatigue disappears by itself after the end of cancer treatment (29%) [9]. Thus, lack of knowledge seems to be associated with false beliefs, detrimental attitudes, anxiety, and distress. It can further be assumed that knowledge gaps regarding the potential manifestations of fatigue, its temporal courses, precipitating, perpetuating, or beneficial factors may hinder adequate prevention as well as treatment of fatigue. On the other hand, a Cochrane review showed that educational interventions focusing on fatigue may contribute to reducing fatigue distress and to alleviating anxiety [11]. Thus, a better patient empowerment, i.e., enabling and encouraging patients to gain the skills and knowledge that will allow them to overcome the burdens of fatigue, is essential.

On the health care professionals’ side, various issues might contribute to this dissatisfactory situation regarding information and education about fatigue such as communication problems, neglecting the syndrome or considering it to be less important, lack of time in medical routine, or insufficient coverage by health insurance [12]. Handing out information material could be an easy, time-, and cost-efficient option. Yet, so far, it was unclear if this measure provides relevant benefits.

Therefore, in this study, we aimed to identify concrete gaps in patients’ knowledge, problematic perceptions, and attitudes regarding fatigue and explore in what way a brief information booklet may be useful in this regard and which aspects might need improvement.

## Methods

### Information booklet

The CARPE DIEM project aimed to contribute to improved health literacy and patient empowerment with regard to fatigue. To this purpose, we developed a brief, easily understandable information booklet with 9 pages covering the topics: (1) What is fatigue? (2) How do I find out that I suffer from fatigue? (3) What are the potential causes of fatigue? and (4) What can I do about my fatigue? The information is based on current evidence and the clinical practice guidelines [2, 3]. To investigate the comprehensibility, benefits, and acceptability of the booklet, a longitudinal study was performed.

### Study population

The study population comprised patients at least 18 years of age, with a first-time diagnosis of any malignant tumor, a current or completed systemic therapy or radiotherapy, who were willing to read the booklet. Insufficient German language skills or inability to read or cognitively comprehend texts were exclusion criteria. Patients were recruited by hand-outs, posters, or direct approach in the outpatient clinic of the National Center for Tumor Diseases (NCT) in Heidelberg.

### Study schedule and procedures

To explore concrete gaps in patients’ knowledge, problematic perceptions, in what way the booklet may be useful and which aspects might need improvement, we set up a questionnaire including items on information, knowledge, perceptions, and attitudes with regard to fatigue as well as questions on the use and evaluation of the booklet (see supplementary file 1). We pre-tested all questions in 10 cancer patients and the knowledge items additionally in 8 healthy volunteers. At baseline (T0), patients were asked to complete the questions on knowledge, perceptions, and attitudes and a validated questionnaire on physical, emotional, and cognitive fatigue (EORTC QLQ-FA12, Cronbach alpha 0.79–0.90 [13]). Thereafter, patients were asked to read the booklet. Additionally, patients were asked to record their general fatigue levels on a scale from 0 = “not tired at all” to 10 = “completely exhausted” as well as their physical, emotional, and cognitive fatigue levels on a 5-point smiley face scale in a symptom diary for 7 days. Participants further indicated sleep duration and sleep quality as well as time spent physically active in the diary. They were then requested to send it back in a prepaid envelope to the study center together with the filled questionnaires (if not completed directly at the study center). One week after the return of the diary and additionally 3 months later, i.e., at a median time of 1 month (T1) and 4 months (T2) after handing out the booklet, patients again received similar questionnaires via postal mail and were asked to complete and return them.

### Assessment of knowledge, perceptions, and attitudes

**Fig. 1:**
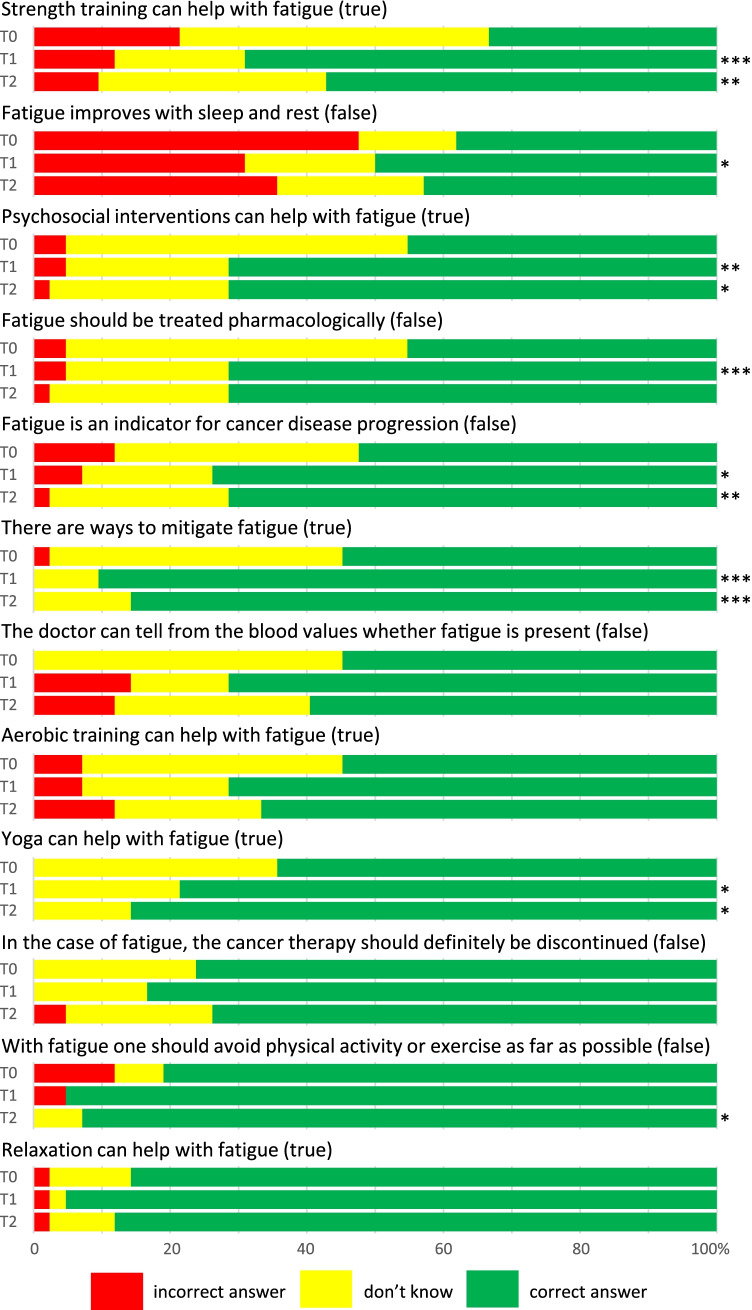
Knowledge about fatigue, stratified by time point. Statements had to be judged by patients as true, false, or “don’t know.” T0: baseline; T1, T2: about 1 and 4 months after handing out the booklet, respectively. *p < .05, **p < .01, ***p < .001 using Wilcoxon signed-rank test for change to baseline

In order to assess knowledge, patients were presented (true or false) statements about fatigue (see Fig. 1) and were asked whether they considered them (rather) true or (rather) false, or if they could not judge them at all (“don’t know”). Perceptions (e.g., feeling helpless in the face of fatigue) and attitudes related to fatigue (e.g., talking with family/friends or physicians about their fatigue) were assessed along a list of several statements that should be rated on a 4-point Likert scale (“fully agree,” “rather agree,” “rather disagree,” “fully disagree”).

### Statistical analysis

Answers to the twelve knowledge questions (as listed in Fig. 1) were summed to a knowledge score (correct: + 1, incorrect: − 1, “don’t know”: 0), with larger scores indicating better knowledge about fatigue. Responses are presented by descriptive statistics. Due to non-normal distribution, differences between T0 and the follow-up timepoints were tested using Wilcoxon signed-rank test. Determinants of knowledge were investigated using analysis of covariance (ANCOVA) models with the knowledge score as dependent variable and age, education, familial situation, BMI, cancer type, and time since diagnosis as independent variables. Due to collinearity, sex was explored in a separate model, excluding cancer type. For parsimony, independent variables that showed no association with the knowledge score and no confounding effects were dropped from the final model. Data on perceptions and attitudes were analyzed descriptively.

## Results

Of 62 patients who signed informed consent and received the study package including the baseline questionnaires, the booklet, and the diary, 4 patients did never reply, 1 deceased before completion, 5 dropped due to temporal stop of the study because of COVID-19 pandemic, 1 due to health reasons, and 1 due to time constraints. Thus, 50 participants provided data and were included in the analyses.

Patients’ characteristics are presented in Table 1. The majority of participants were younger than 55 years (62.0%) and female (84.0%), 66.0% were diagnosed with breast cancer, and 62.0% received chemotherapy. Except for 4 patients who had already completed their cancer treatment, all participants were on ongoing systemic or radiotherapy. More than a third (36.0%) had been diagnosed with cancer at least 12 months ago. Baseline physical fatigue (median (Q1, Q3) = 53 (27, 80)), emotional fatigue (33 (11, 44)), and cognitive fatigue (17 (0, 33) levels were clearly above those of the general German population (20 (0, 46), 0 (0, 22), and 0 (0, 17) respectively, for women age 40–57 years; scores for males even lower [14]).

**Table 1:** Patient characteristics at baseline

Characteristics	Study population (*n* = 50)		
Sex	Female	42	84.0%
	Male	8	16.0%
Age	Mean (SD)	54.3	(13.7)
	≤ 45 years	14	28.0%
	≤ 55 years	17	34.0%
	≤ 65 years	7	14.0%
	≤ 75 years	8	16.0%
	> 75 years	4	8.0%
Body mass index	< 25	21	42.0%
	25– < 30	19	38.0%
	≥ 30	10	20.0%
School degree	University-entrance diploma^a^	22	44.0%
	High school degree^b^	12	24.0%
	Secondary school degree^c^	14	28.0%
	Missing	2	4.0%
Cancer type	Breast cancer	33	66.0%
	Skin cancer	9	18.0%
	Uveal cancer	2	4.0%
	Other^d^	6	12.0%
Treatment	Chemotherapy	31	62.0%
	Radiotherapy	18	36.0%
	Endocrine therapy	8	16.0%
	Immune therapy	15	30.0%
	Ongoing	46	92.0%
	Completed	4	8.0%
Metastases	No	30	60.0%
	Yes	20	40.0%
Time since diagnosis	12 + months	18	36.0%
	< 12 months	13	26.0%
	< 6 months	14	28.0%
	Missing	5	10.0%
Physical fatigue^e^	Median (Q1, Q3)	53	(27, 80)
Emotional fatigue^e^	Median (Q1, Q3)	33	(11, 44)
Cognitive fatigue^e^	Median (Q1, Q3)	17	(0, 33)

^a^German “(Fach-)Abitur”

^b^German “Mittlere Reife”

^c^German “Hauptschulabschluss”

^d^Other: each *n* = 1 of lymphoma, gastric, pancreatic, lung, neuroendocrine, and endometrial cancer

^e^Assessed with the multidimensional EORTC QLQ-FA12 questionnaires; 0–100 scale

In everyday German language, the word “fatigue” does not exist, yet it is the medical term commonly used in Germany in clinical practice and research for this syndrome. There is no layman’s term for this. At baseline, 27.1% had not heard of the medical term “fatigue” before, while 10.4% had heard the term but did not know what it meant (Table 2). In contrast, 25.0% reported being very well informed about fatigue, which were mainly patients having received higher education. Among the patients that reported to be at least somewhat or very well informed about fatigue, the majority had read about it in booklets, books, or articles (63.3%) and/or on the internet (46.7%). Only 30.0% of the informed patients got their information about fatigue from the treating physician. Hence, also taking the uninformed patients into account, this results in only 9 of 48 patients (18.8%) being informed by the treating physician about fatigue. Information by nurses (8.3%) or general practitioners (4.2%) was even rarer.

**Table 2:** State of knowledge and information at baseline, stratified by education

	TOTAL		University-entrance diploma^a^		High school degree^b^		Secondary school degree^c^	
	*N*	%	*N*	%	*N*	%	*N*	%
**Does the term “fatigue” have any meaning to you?**^d^ (*n* = 48) *P* < .0001*								
No, never heard	13	27.1	3	13.6	3	25.0	7	50.0
Already heard, but do not know what it means	5	10.4	2	9.1	2	16.7	1	7.1
I have already heard or read a little about it	18	37.5	8	36.4	6	50.0	4	28.6
I am very well informed about it	12	25.0	9	40.9	1	8.3	2	14.3
If the term “fatigue” is known (*n* = 30): **Where did you get your information about fatigue?** (multiple answers possible)								
Booklets, books, publications	18	63.3	10	58.8	5	71.4	4	66.7
Internet	14	46.7	10	58.8	2	28.6	2	33.3
Treating physician	9	30.0	5	29.4	4	57.1	0	0.0
Other patients	6	20.0	4	23.5	2	28.6	0	0.0
Nurses	4	13.3	2	11.8	1	14.3	1	16.7
Information events	3	11.8	2	11.8	0	0.0	1	16.7
General practicioner	2	6.7	1	5.9	1	14.3	0	0.0

^*^Fisher’s exact test for differences between educational levels

^a^German “(Fach-)Abitur”

^b^German “Mittlere Reife”

^c^German “Hauptschulabschluss”

^d^ “Fatigue” is the medical term commonly used also in Germany for this syndrome, but it is not a word in the everyday German language

The majority of participants stated having read the booklet completely including the “Read More” parts (63.0%) or almost completely (34.8%). Hereby, more patients with lower education had read it completely (84.6%) compared to those with a middle or high education (54.5%). Irrespective of education, nearly all patients (95.6%) reported that they would recommend the booklet to others (data not shown).

Figure 1 shows the proportions of correct, incorrect, or “don't know” answers to the knowledge questions at T0, T1, and T2. Regarding potentially beneficial treatment approaches, there were major knowledge gaps at T0 with respect to strength training (66.7% incorrect or don’t know) and psychosocial interventions (54.8%), but several patients also did not know about aerobic training (45.2%) or yoga (35.7%) as potentially beneficial approaches. At T1, the knowledge significantly improved regarding most of the statements. About 4 months after handing out the booklet (T2), significant improvements compared to T0 were still seen for several items.

ANCOVA models revealed that lower knowledge scores at baseline were significantly associated with higher age (*p* = 0.0084) and lower education (*p* < 0.0001). Sex, cancer type, metastases, type of cancer therapy, time since the first diagnosis, familial status, BMI, or presence of fatigue were not significantly associated with knowledge. The distribution of the knowledge score, stratified by education, is shown in Table 3. Overall, the knowledge score increased significantly from T0 to T1 and T2.

**Table 3:** Distribution of the knowledge score, stratified by education

	**TOTAL**			University-entrance diploma^a^		High school degree^b^		Secondary school degree^c^	
	*n*	Median	(Q1, Q3)	Median	(Q1, Q3)	Median	(Q1, Q3)	Median	(Q1, Q3)
T0	48	6.0	(3.0, 9.0)	8.0	(6.0, 10.0)	7.0	(4.0, 8.5)	2.0	(− 1.0, 4.0)
T1	48	10.0	(7.0, 11.0)***	10.0	(9.0, 11.0)**	10.5	(8.5, 12.0)**	6.0	(3.0, 8.0)***
T2	44	8.0	(6.0, 10.5)***	10.0	(8.0, 11.0)	9.0	(7.0, 10.0)*	5.0	(3.0, 6.5)**

Difference to T0, Wilcoxon sign rank test: *p < .05, **p < .01, ***p < .001 (separately for each education level)

^a^German “(Fach-)Abitur”

^b^German “Mittlere Reife”

^c^German “Hauptschulabschluss”

After reading the booklet (T1), fewer patients felt helpless in the face of fatigue (23.1% vs. 46.1%) or poorly informed about fatigue (12.8% vs. 56.4%) compared to T0 (data not shown). Patients’ ratings about the impact and the usefulness of the booklet, assessed at T1, are summarized in Table 4. Almost all patients fully or rather agreed that the booklet taught them new things about fatigue (91.1%). Among those patients who ever had experienced fatigue, the vast majority (89.7%) reported that the booklet made them realize that they are not alone with their problems and helped them to find a suitable measure against fatigue (72.9%). Moreover, it encouraged them to talk with health care professionals (52.7%) and family and friends (55.3%). Regarding potential negative effects of the booklet, 9.3% rather agreed that the booklet triggered or intensified fears and concerns about the side effects of the cancer treatment.

**Table 4:** Patient-reported impact of the information booklet

The booklet…	**Fully agree**		**Rather yes**		**Rather no**		**Not at all**	
	***n***	**%**	***n***	**%**	***n***	**%**	***n***	**%**
…made me realize that I am not alone with my problems^a^	24	61.5	11	28.2	0	0.0	4	10.3
…taught me new things about fatigue	22	48.9	19	42.2	3	6.7	1	2.2
…helped me to find a suitable measure against my fatigue^a^	9	24.3	18	48.6	7	18.9	3	8.1
…encouraged me to talk with a doctor or other medical/psychological professionals^a^	5	13.2	15	39.5	13	34.2	5	13.2
…encouraged me to talk to family and/or friends about my fatigue^a^	3	7.9	18	47.4	12	31.6	5	13.2
…triggered or intensified fears and concerns about side effects of the cancer treatment	0	0.0	4	9.3	10	23.3	29	67.4

^a^Excluding 6 patients who reported never having any exhaustion in the course of cancer treatment

## Discussion

The CARPE DIEM study revealed specific knowledge gaps and open needs with respect to fatigue among cancer patients, especially among those with lower education or higher age. The results suggest that handing out and motivating to read a brief information booklet can significantly improve knowledge and attitudes.

The finding that more than a third of the enrolled cancer patients did not know the term “fatigue” or what it means confirms our experiences from communication with patients in the day-to-day clinical routine. Even patients suffering from fatigue symptoms often do not know that their burden has a name and is a common phenomenon. In a recent large survey in 2508 cancer survivors about 2 years after diagnosis [9], a similarly high proportion (58%) reported feeling not well informed about fatigue as in the CARPE DIEM study (56%). This deficiency of information is a fundamental problem, which can hinder patients’ access to preventive measures and prevent them from seeking help and getting treatment for their problems.

Regarding specific knowledge aspects, almost half of the patients did not know that fatigue is generally not a sign of cancer progression. Stress and anxiety due to misinterpreted fatigue symptoms could be reduced by providing early and understandable information and by reassuring patients and family members that fatigue is not necessarily an indicator for disease progression. This is explicitly recommended by the NCCN guidelines [2]. Nevertheless, treating physicians should evaluate among others also the disease status when patients report about the new onset of extreme exhaustion.

Furthermore, there were large gaps in knowledge regarding treatment options for patients with fatigue. Current guidelines recommend aerobic and strength exercise of moderate intensity with the highest level of evidence as well as general physical activity, yoga, and psycho-social interventions [2, 3]. Relaxation techniques can also be beneficial, especially in elderly cancer patients [3]. Non-pharmacological therapies significantly outperform pharmacological treatments [4]. Accordingly, pharmacological interventions are not recommended in general. Only in patients with advanced disease methylphenidate [2] or glucocorticoids [2, 3] might be considered if non-pharmacological therapies failed. Our results revealed a lack of knowledge about these potential fatigue treatment options in about two-thirds of patients, especially regarding strength training and psycho-social interventions, but to a lesser extent also regarding aerobic exercise and yoga. These results underpin previous findings that treatment options were too rarely taken up by patients with fatigue [9].

Additionally, there is consensus that commonly physical inactivity should be avoided in case of cancer-related fatigue and that inactivity and too much resting may contribute to a decline in physical fitness and increased fatigue [2, 3]. However, some patients believed that physical activity and exercise should be avoided when fatigued or were unclear about the benefits of physical activity. Moreover, there was uncertainty in many patients as to whether fatigue is relieved by sleep and rest. As a consequence, patients might adopt detrimental behaviors, i.e., too much resting and reducing physical activity. It has to be noted, nevertheless, that fatigue in some cancer patients seems to be associated with post-exertional malaise, i.e., a worsening of symptoms after physical or mental activity [15, 16]. More research on potential fatigue subtypes is needed to provide more specific recommendations regarding physical activity for these patients.

One month after handing out the information booklet, the proportion of patients knowing that there are options to ameliorate fatigue increased from 55% to 90% and remained at a high level even after 4 months. Therefore, although an information booklet certainly cannot solve the fatigue problems completely, it can at least increase health competency in several patients and may encourage them to seek further support. Overall, the proportion of patients feeling poorly informed significantly decreased. Educating about fatigue seemed to also reduce emotional burden in patients: First, the booklet made most patients realize that they are not alone with their problems. This was perceived as helpful by several patients, who often had struggled with the fact that their fatigue was not taken seriously by others. Second, after providing information about fatigue, the number of patients feeling helpless in the face of fatigue decreased. Third, it encouraged about half of the participants to talk about their fatigue with health care professionals as well as with family and friends. On the other hand, information at the beginning of cancer therapy about potential adverse effects may evoke worries. This was, however, rare in our study.

Our findings add to the currently still limited evidence regarding the effects of education on managing cancer-related fatigue. A Cochrane review found a small reduction in fatigue intensity derived from 8 RCTs (*n* = 1524, standardized mean difference (SMD): − 0.28 (95% CI: − 0.52 to − 0.04)), a small reduction in fatigue interference with daily life (SMD: − 0.35 (95% CI: − 0.54 to − 0.16), 4 studies, *n* = 439) as well as in anxiety (mean difference: –1.47 (95% CI: − 2.76 to − 0.18), 3 studies, *n* = 571) comparing educational interventions with usual care or attention control [17]. A recent meta-analysis in breast cancer survivors post-treatment also showed a small reduction in fatigue (SMD: − 0.24 (− 0.37 to − 0.11)) comparing patient education versus usual care [18]. Even these effect sizes are not huge, those of the most promising approaches for the treatment of fatigue, i.e., exercise, yoga, psychosocial and mindfulness-based interventions, are commonly also only small to moderate, indicating that there is likely no single one-fits-all approach to ameliorate fatigue. Thus, providing information about fatigue could (and should) be considered as a simple common step in the care of cancer patients besides other additional approaches.

Patients who were not completely uninformed about fatigue at study enrolment had derived their knowledge about fatigue mainly from booklets, articles, or the internet on their own initiative. Only 19% of participants were adequately informed about fatigue by the treating physician and even fewer by nurses or general practitioners. This finding is in line with the large FiX survey (*n* = 2508), where 41% of cancer survivors reported having never been asked about being exhausted by their treating physician [9]. Lack of education and counseling on fatigue was also observed in other studies outside Germany [19, 20]. In a US survey of 2487 patients with breast or colon cancer, 61% reported having talked about fatigue with a physician and only 40% got the help they wanted [21].

What might be barriers to informing about fatigue or to providing information materials? Likely, there are multiple reasons. While according to the current clinical standard, the treating physician is in charge of informing the patient about possible side effects of the planned cancer therapy, there are no clear instructions on when, how, and by whom fatigue should be addressed in more detail. Recent research further mentioned health care professionals’ lack of knowledge about fatigue and the treatment options, the interdisciplinary nature of fatigue guidelines and management, time and funding constraints, as well as gaps in the patient–provider communication [12, 22, 23]. Health care professionals sometimes also seem to underestimate the problem or to consider it to be secondary to other symptoms such as pain [24]. Training of health care and community support providers as well as specific knowledge translation tools have been shown to increase knowledge and intentions to implement recommendations and, therefore, would be one step in overcoming this problem [25].

Even if information materials such as booklets or hand-outs exist, patients might not notice or read them because they are often already overwhelmed with information regarding medical, practical, regulatory, and financial issues. For some patients, language or cognitive skills might be a barrier to understand the provided oral or written information. Finally, patients with depressive symptoms or emotional distress might lack the drive to read a booklet. However, just these patients are at high risk for fatigue. Hence, providing information materials about fatigue free of charge in the cancer centers or treating facilities should be a standard, but this does not exempt health care professionals from the responsibility to address and explain fatigue in person to patients.

Baseline knowledge about fatigue was significantly lower in patients with lower education or higher age. Less educated patients more often had not heard about fatigue before study enrolment. This confirms previous findings regarding general health literacy [26]. Since informed patients had gotten their knowledge mainly by reading booklets or researching on the internet on their own initiative, patients with lower education might lack the motivation, time, or ability to do this. Therefore, patients with lower education probably should be educated about fatigue personally in an understandable way or be informed by directly handing out easy-to-understand information material. We had designed our booklet with brief, easy-to-read texts enlivened by photos and pictures. Almost all study participants with lower education stated having read the booklet completely.

Social inequalities and cultural or language barriers could not be investigated with our data, but it should not remain unmentioned that these are commonly major reasons for inadequate health literacy [26] and likely also play a role in knowledge and empowerment with respect to fatigue. Thus, it would be helpful to provide information material, at least for the most common immigrant groups in their native languages.

The primary aim of the CARPE DIEM study was to evaluate and optimize an information booklet and a diary. Therefore, the study was not randomized which is a limitation for the actual analysis regarding the impact of the booklet on knowledge. The knowledge questionnaire also has some limitations as some statements are not entirely unambiguous. For example, the statement “fatigue is an indicator for cancer disease progression” could be true in some cases, but having fatigue commonly does not mean that the cancer is progressing. “Fatigue should be treated pharmacologically” might be considered correct with respect to disorders that contribute to symptoms of fatigue (e.g., anemia, endocrine disorders). Yet, for cancer-related fatigue in the narrower sense, the guidelines generally do not recommend pharmacological treatment (except for some rarer cases). Overall, as we asked the participants to mark the answer (true/false) they think is most appropriate, it may be assumed that the statement was mostly interpreted correctly. Furthermore, there may be a selection bias, as likely only patients who are generally willing to read a booklet consented to participate and returned the questionnaires. However, it does not diminish the value and usefulness of information material if it is not evaluated among patients who are unable and generally not willing to read it. For these individuals, other outreach strategies need to be developed to improve their knowledge about fatigue and their health literacy. The positive study results and patients’ feedback suggest that an easy-to-read booklet may be a useful offer for many patients.

## Conclusion

The CARPE DIEM study identified a number of specific gaps in the provision of information and education for cancer patients about fatigue. A low-level intervention asking to read a brief and easily comprehensible booklet contributed to increased health literacy. This could be a cost-effective measure to improve fatigue management. However, it should only be considered as a first step offered as part of a bundle of further efforts to improve knowledge and fatigue care.

## Supplementary Information

Below is the link to the electronic supplementary material.

Supplementary file1 (PDF 259 KB)

## Data Availability

Data can be made available to scientific cooperation partners on request.

## Code availability

N/A.
