# Supplementary material for: Cancer-related fatigue: benefits of information booklets to improve patients’ knowledge and empowerment
Source: Support Care Cancer. 2022 Feb 11;30(6):4813–21. doi: 10.1007/s00520-022-06833-w (PMC8853058; doi:10.1007/s00520-022-06833-w)
Supplement: Supplementary file 1 — Supplementary file1 (PDF 259 KB) [file 520_2022_6833_MOESM1_ESM.pdf]

Attachment: Excerpts from the CARPE DIEM study questionnaires including the aspects addressed in the manuscript (translated from German)

- *the assessment timepoints for each question are indicated in brackets*
- *t0 = baseline; t1 = about one month after reading the information booklet; t2 = about four months after reading the information booklet*
- *numbering of the questions doesn't correspond to the original questionnaires*

1. Have you ever heard of the term "fatigue"? *[t0]*

- ☐ No, I have never heard of it. → *continue with question 2.*
- ☐ I have heard of it, but I actually don't know what it means. → *continue with question 2.*
- ☐ I have somewhat heard of it or read about it.
- ☐ I am very well informed about it.

1.1 Where did you get your information about fatigue? *(multiple answers are possible)*

- ☐ from my treating physicians
- ☐ from my general practitioner
- ☐ from nurses
- ☐ from other patients
- ☐ I have read about it on the internet.
- ☐ I have read about it in booklets, books or articles.
- ☐ from informative meetings
- ☐ other sources of information → *which ones?* ____________________

2. Which of the following statements do you consider as true or false? *[t0, t1, t2]*

If you are not completely sure, please tick the answer that you consider as most likely to be appropriate.

		true	false	can't judge it at all
1.	Strength training can help with fatigue.	☐	☐	☐
2.	Fatigue improves with sleep and rest.	☐	☐	☐
3.	Psychosocial interventions can help with fatigue.	☐	☐	☐
4.	Fatigue should be treated pharmacologically.	☐	☐	☐
5.	Fatigue is an indicator for cancer disease progression.	☐	☐	☐
6.	There are ways to mitigate fatigue.	☐	☐	☐
7.	The doctor can tell from the blood values whether fatigue is present.	☐	☐	☐
8.	Aerobic training can help with fatigue.	☐	☐	☐
9.	Yoga can help with fatigue.	☐	☐	☐
10.	In the case of fatigue, the cancer therapy should definitely be discontinued.	☐	☐	☐
11.	With fatigue one should avoid physical activity or exercise as far as possible.	☐	☐	☐
12.	Relaxation can help with fatigue.	☐	☐	☐

3. Do you feel extremely exhausted since cancer diagnosis/ treatment? *[t0,t1,t2]*

☐ never/rarely
☐ sometimes
☐ often → *please continue with question 3.1*
☐ always

3.1 To what extent do the following statements apply to you personally?

		fully disagree	rather disagree	rather agree	fully agree
1.	I feel helpless in the face of fatigue.	☐	☐	☐	☐
2.	I feel well informed about exhaustion/fatigue.	☐	☐	☐	☐

4. Questions regarding content and layout of the information booklet. *[t1]*

For the following questions please tick the answer that best applies to you.

1.	How did you find the layout of the information booklet?	☐ very good<br>☐ good<br>☐ okay<br>☐ bad<br>☐ very bad
2.	How did you experience reading the texts?	☐ very arduous<br>☐ arduous<br>☐ enjoyable/interesting<br>☐ okay<br>☐ boring
3.	How did you judge the information content?	☐ too detailed<br>☐ a bit too detailed<br>☐ appropriate<br>☐ a bit too superficial<br>☐ too superficial
4.	Did you read the whole information booklet?	☐ Yes, I read it completely (including “further information”).<br>☐ Yes, I read almost the whole booklet.<br>☐ No, I read about half of the booklet.<br>☐ No, I read less than half of the booklet.
5.	Would you recommend the information booklet?	☐ yes<br>☐ no

5. **Which chapters of the information booklet did you find useful and/or interesting?** *[t1]*

		Chapter was useful/interesting			
		very much	partly	somewhat	not at all
1.	Chapter 1: “What is fatigue?” (p.3-4)	☐	☐	☐	☐
2.	Chapter 2: “How do I find out that I suffer from fatigue?” (p. 5)	☐	☐	☐	☐
3.	Chapter 3: “What are potential causes of fatigue?” (p. 6)	☐	☐	☐	☐
4.	Chapter 4: “What can I do about my fatigue?” (p. 7)	☐	☐	☐	☐
5.	Supplementary Chapter: “Further information” (p. 8-10)	☐	☐	☐	☐

6. **To what extent was the information booklet useful for you?** *[t1]*

		fully disagree	rather disagree	rather agree	fully agree
1.	The information booklet encouraged me to talk to family and/or friends about my exhaustion.	☐	☐	☐	☐
2.	The information booklet helped me to find a suitable measure against exhaustion.	☐	☐	☐	☐
3.	The information booklet encouraged me to talk to a physician or to other health care professionals about my exhaustion.	☐	☐	☐	☐
4.	The information booklet triggered or intensified fears or concerns about side effects of cancer treatment.	☐	☐	☐	☐
5.	The information booklet taught me new things about fatigue.	☐	☐	☐	☐
6.	The information booklet made me realize that I am not alone with my problems.	☐	☐	☐	☐

Further comments or suggestions concerning the information booklet: *[t1]*
